# Selenium in Prostate Cancer: Prevention, Progression, and Treatment

**DOI:** 10.3390/ph16091250

**Published:** 2023-09-05

**Authors:** Jinjiang Jiang, Bo Chen, Bo Tang, Qiang Wei

**Affiliations:** 1 Department of Urology, West China Hospital of Sichuan University, No. 37, Guoxue Lane, Chengdu 610041, China; 2 Institute of Urology, West China Hospital of Sichuan University, Chengdu 610041, China

**Keywords:** prostate cancer, selenium, selenoprotein, chemoprevention, therapy, progression

## Abstract

Selenium, a trace mineral with various biological functions, has become a focal point in prostate cancer research. This review aims to present a comprehensive overview of selenium’s involvement in prostate cancer, covering its impact on prevention, development, treatment, and underlying mechanisms. Observational studies have revealed a link between selenium levels and selenoproteins with prostate cancer progression. However, randomized controlled studies have shown that selenium supplementation does not prevent prostate cancer (HR: 0.95; 95% CI 0.80–1.13). This discrepancy might be attributed to selenoprotein single nucleotide polymorphisms. In the context of combinatorial therapy, selenium has demonstrated promising synergistic potential in the treatment of prostate cancer. Emerging evidence highlights the significant role of selenium and selenoproteins in prostate cancer, encompassing AR signaling, antioxidative properties, cell death, cell cycle regulation, angiogenesis, epigenetic regulation, immunoregulation, epithelial–mesenchymal transformation, and redox signal. In conclusion, selenium’s diverse properties make it a promising trace mineral in prostate cancer prevention, development, and treatment and as a platform for exploring novel agents.

## 1. Introduction

Prostate cancer is ranked second among male malignancies worldwide and holds the top spot in the USA [1]. Its development and progression are influenced by a range of genetic and environmental factors [2,3]. A recent study has shown that making significant lifestyle changes can have an impact on the advancement of low-grade and early-stage prostate cancer [4]. For example, using 5-alpha reductase inhibitors [5], consuming green tea catechins [6], phytoestrogen [7], and carotenoids [8] have been found to affect the progression of the disease. Additionally, the intake of certain minerals, particularly zinc, calcium, and selenium, along with high consumption of dietary isoflavones, is recognized as factors that can influence the risk of prostate cancer [9]. Among the many dietary components and micronutrients that have been investigated in relation to prostate cancer risk, selenium has emerged as a promising element with potential implications for both prevention and treatment.

Selenium, which is an essential trace mineral, has undergone extensive research regarding its correlation with prostate cancer. These studies have delved into its role in antioxidant defense [10], androgen receptor signaling [11], cell death [12], cell cycle [13], and angiogenesis [14]. By gaining a deeper understanding of how selenium impacts the development and progression of prostate cancer, we can gain valuable insights into its potential effects on disease advancement, progression, and therapeutic approaches.

Moreover, there is increasing evidence suggesting that selenium can serve as a preventive agent, and the levels of selenium in the bloodstream may be linked to the development of prostate cancer [15]. Interestingly, both low and high levels of selenium have shown potential implications. Furthermore, recent research has uncovered that genetic factors and single nucleotide polymorphisms (SNPs) in selenoproteins [16] can influence how the body responds to selenium in preventing prostate cancer. Understanding the impact of genetic background on prostate cancer can enrich our understanding and facilitate the development of targeted strategies for its prevention and management.

In light of this, our goal is to bring some insights regarding the role of selenium in prostate cancer and stimulate further research in this field.

## 2. Absorption, Distribution, Metabolism, and Excretion (ADME) of Selenium

**Figure 1:**
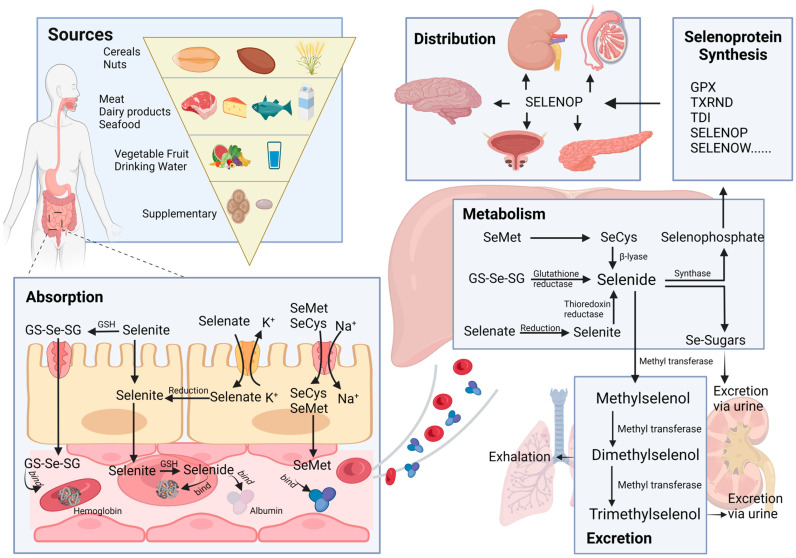
Schematic representation of selenium’s biological process in the human body.

The way selenium moves through the body is a complex interplay of various mechanisms. The processes of absorption, distribution, metabolism, and elimination (ADME) are the key players in this journey, as illustrated in Figure 1. Grasping these processes is crucial for evaluating the effectiveness and safety of using selenium in therapeutic applications.

### 2.1. Sources

Selenium enters the human body through various sources, including food, supplements, and drinking water. In these sources, selenium primarily exists in the forms of selenomethionine (SeMet), selenocysteine (SeCys), methylselenocysteine, sodium selenite, and selenate [17]. Inorganic forms, such as selenite and selenate, exist in plants and undergo conversion to SeMet and SeCys. These organic forms of selenium serve as the primary source of selenium intake from plant-based foods, which encompass a wide variety of items, such as cereals, nuts, vegetables, and fruits. Among these, cereals hold particular significance as a primary source of selenium [18]. Kieliszek et al. reported that cereal products contribute to approximately 50% of the daily total selenium intake in the Spanish population [19]. On the other hand, fruits and vegetables are not abundant sources of selenium [20], and only a small portion of them can meet the body’s selenium demand. Animal-based foods such as meat, dairy products, fish, seafood, and milk are rich sources of selenium in our daily diet [19] and are widely recognized as another essential source, complementing the contribution of cereals. The dominant forms of selenium found in these animal-based foods are SeMet and SeCys.

Besides the selenium naturally present in foods, various selenium supplements have been developed to meet the body’s need for trace elements. These supplements include selenite, selenate, selenium yeast, selenium-enriched edible fungus powder, and selenized carrageenan [20]. Lastly, selenium can also be found in water, primarily in the forms of selenate and selenite. 

### 2.2. Absorption

The absorption of selenium and its compounds takes place in the duodenum and small intestine, facilitated by various transport mechanisms, with varying absorption efficiencies in different forms of selenium. While elementary selenium, selenium dioxide, and selenium sulfide are challenging to absorb, selenites, selenates, and selenium-containing amino acid derivatives are more readily absorbed, especially in the presence of vitamins A, D, and E [21]. Selenate is primarily transported via the Na^+^/K^+^/Cl^−^ cotransporter or OH^−^ antiporter, selenite is transported through Na^+^-independent passive transport, and selenium-containing amino acids, such as SeMet and SeCys, are absorbed through specialized Na^+^-dependent amino acid transporters [22,23]. 

### 2.3. Distribution 

Different forms of selenium are absorbed through the epithelium of the small intestine and subsequently enter the liver via the hepatic portal vein, where initial selenium metabolism occurs. Direct absorption of selenite does not exceed 60%, with the majority of it undergoing reduction to selenodiglutathione (GS-Se-SG) in the intestinal lumen, promoting better absorption [24]. A minor portion of selenite and GS-Se-SG is metabolized to selenide, which selectively binds to albumin and hemoglobin [25] and is then transported to the liver. SeMet is also transported to the liver facilitated by albumin after absorption. However, the delivery process of Selenate and SeCys remains unclear. Selenate is reported to undergo conversion to selenite through ATP-sulfurylase, while SeCys could be transformed into SeMet [26]. It is plausible that selenate and SeCys can be transformed into forms that can bind to plasma proteins.

Selenoprotein P (SELENOP), produced by the liver, is secreted into the plasma to deliver selenium to peripheral tissues [27]. The local uptake of selenium from plasma is mediated by receptors. SELENOP binds to apolipoprotein E receptor-2 (ApoER2) in seminiferous epithelium and neurons [28,29]. Additionally, Olson et al. reported that the lipoprotein receptor megalin facilitates the uptake of SELENOP from the glomerular filtrate into the proximal tubule epithelial cells of the kidneys [30]. However, the mechanism of selenium uptake by prostatic epithelium is unclear, and the specific binding receptor for the transporter molecule requires further investigation.

### 2.4. Metabolism

All forms of Se can undergo metabolism to selenide (H2Se), which plays a crucial role in selenoprotein synthesis and the methylation excretion of selenium. As previously mentioned, selenite can be converted to GS-Se-SG in the presence of reduced glutathione (GSH), and subsequently, GS-SE-SG can be further metabolized to H2Se. Additionally, selenite can act as a substrate for the thioredoxin (TXRND) system and directly be reduced to H2Se [24]. Selenate, on the other hand, is reduced to selenite through ATP sulfurylase during sulfate reduction. Moreover, SeMet can be transformed into SeCys, and SeCys can be converted to H2Se via the trans-selenation pathway [31]. H2Se is incorporated into selenoproteins, including glutathione peroxidases (GPX), iodothyronine deiodinases (TDI), TXRND, selenophosphate synthetase (SePsyn), SELENOP and selenoprotein W (SELENOW), and Selenocysteinyl-tRNA, a crucial transport RNA, to synthesize selenoprotein [26].

### 2.5. Excretion

In situations of excessive selenium status, selenium is typically converted into methylated forms. In this process, SeMet and H2Se undergo a stepwise methylation process, leading to the formation of methylselenol. Further metabolic conversion of methylselenol results in the production of dimethyl selenide, which is subsequently eliminated through respiratory efflux, while trimethyl selenonium is excreted through the urine [32,33]. Conversely, under conditions of low selenium levels, H2Se is transformed into selenosugars, which are then excreted in the urine [34].

## 3. Evidence from Epidemiological Studies

### 3.1. Observational Studies of Selenium and Prostate Cancer

Selenium and selenoprotein levels have shown associations with the risk of various human tumors, such as thyroid tumors [35], ovarian cancer [36], and breast cancer [37]. Generally, lower serum selenium status has been correlated with an increased risk of cancer. However, there is no significant association between plasma SELENOP concentration and lung cancer risk among Shanghai men [38]. These findings indicate that the effects of selenium proteins may vary depending on the specific tissue or organ.

Numerous investigations have explored the association between serum selenium levels and the risk of prostate cancer. In a meta-analysis by Cui et al. [39], which incorporated 17 studies measuring selenium status through serum and toenail selenium, an inverse relationship between serum selenium levels and prostate cancer risk was observed (Odds Risk = 0.72, 95%CI: 0.61–0.86). The analysis of a linear dose–response relationship revealed that an increase of 10 μg/L in serum selenium and 0.1 μg/g in toenail selenium were correlated with a corresponding change in prostate cancer risk. 

Current investigation [40] collected plasma samples from 116 Caucasian men diagnosed with late-onset prostate cancer and 132 matched controls in South Australia, revealing that the mean plasma selenium concentration in prostate cancer cases was significantly lower compared to controls. This finding confirms the abnormal selenium levels observed in prostate cancer cases. Notably, this study also revealed elevated levels of other elements such as iron, copper, calcium, and sulfur in prostate cancer cases, implying a potential synergistic anti-cancer effect of trace elements.

In contrast, results from the Danish “Diet, Cancer and Health” cohort, comprising 784 incident prostate cancer cases and controls, demonstrated comparable median plasma selenium and SELENOP concentrations in both groups, with higher selenium levels observed in high-grade prostate cancer cases [41]. These indicated that plasma selenium status was not associated with the overall risk of prostate cancer, but higher selenium levels were associated with a lower risk of high-grade prostate cancer. This is consistent with the conclusions from Xie et al. [15], which demonstrated that genetic polymorphisms in selenoproteins influence serum selenium levels and are linked to the risk of aggressive prostate cancer in men. Taken collectively, it is plausible that selenium may play a role in the progression of prostate cancer rather than its initiation. Gerstenberger and his colleague [42] analyzed the plasma selenium concentrations of 568 patients with non-metastatic prostate cancer and revealed that the recurrence rate of prostate cancer remained unaffected despite selenium concentration increase, suggesting that selenium might not exert an impact on metastatic events. 

Above these, it seems that selenium probably plays a role in the progression of prostate cancer rather than its initiation or metastasis events. 

### 3.2. Chemoprevention of Selenium and Prostate Cancer

The field of selenium research and its impact on prostate cancer chemoprevention is continuously advancing, with ongoing investigations on selenium supplementation for prevention, as well as the potential synergistic effects of other anti-cancer compounds. Herein, we have reviewed clinical trials, to date, of selenium supplementation for prostate cancer prevention in Table 1. These research studies have explored whether selenium supplementation could prevent prostate cancer in different populations, including the general population [43,44] and those at high risk for prostate cancer, such as HGPIN and men with a history of non-small-cell lung cancer [45,46,47]. 

**Figure 2:**
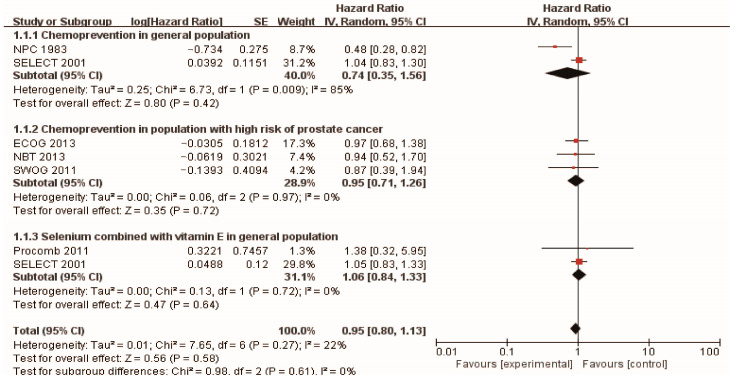
Forest plot showing selenium chemoprevention’s property with or without vitamin E in the general population and population with high risk of prostate cancer. Oxidants (with and without selenium) by smoking and health status and all-cause mortality.

In the context of prostate cancer prevention trials conducted in healthy populations, the Nutritional Prevention of Cancer (NPC) Trial [43] was carried out in 1983, with 457 men randomly assigned to receive selenium supplementation and followed for 13 years. The results indicated that a daily supplement of 200 mg of high selenium yeast could reduce the risk of prostate cancer (HR 0.48, 95% CI 0.28–0.80). Additionally, the SELECT trial [44] recruited 35,533 healthy men with normal digital rectal examination and PSA levels no higher than 4.0 ng/mL, and the participants were randomly assigned to four groups: L-selenomethionine (200 µg/d), vitamin E (400 IU/d), L-selenomethionine + vitamin E, and placebo. After a median follow-up of 5.46 years, the study found that daily selenium yeast supplementation did not exhibit any preventive influence on prostate cancer. Surprisingly, the combination of selenium and vitamin E even demonstrated a harmful effect on the incidence of prostate cancer. The findings from these two cohort studies have sparked some controversy. However, it is worth noting that both studies were based on extensive data from large populations, which lends weight to the reasonability and reliability of their results. To gain a more precise understanding of the preventive impact of selenium supplementation on prostate cancer, we meticulously synthesized the findings. Ultimately, our analysis revealed that selenium supplementation did not show any preventive effect on the development of prostate cancer in healthy men (HR 0.74, 95% CI 0.35–1.56), which is presented in Figure 2.

Numerous studies have explored the potential of selenium supplementation in reducing the risk of prostate cancer among high-risk men. High-grade prostatic intraepithelial neoplasia (HGPIN) is considered a preliminary stage or precancerous form of prostate cancer [48], and initial research suggested that HGPIN detected on core biopsy might indicate a higher likelihood of invasive cancer on repeat biopsy [49]. In the longitudinal SWOG study [45], 423 patients with HGPIN were randomly assigned to either a selenomethionine supplement group or a control group and were followed up for 3 years. The study revealed that the incidence of prostate cancer was 36.6% in the placebo group and 35.6% in the selenium group, showing no significant difference between the two groups. These findings are consistent with another study investigating selenium supplementation for chemoprevention in high-risk populations. The hazard ratios for the risk of developing prostate cancer in the groups taking 200 µg/day or 400 µg/day of selenium tablet were 0.94 [0.52, 1.7] and 0.90 [0.48, 1.7], respectively. Additionally, the rate of PSA velocity in the selenium groups did not significantly differ from the placebo group. These results suggest that neither of the two doses of selenium substantially impacted prostate cancer prevention.

Furthermore, foundational studies have proposed that antioxidants, such as vitamin E and lycopene [50], may enhance the effectiveness of selenium in preventing the formation of mammary tumors. However, in the SELECT study, the combination of vitamin E and selenium demonstrated a negative impact on the onset of prostate cancer. Additionally, in the Procomb study [43], Giuseppe et al. administered 134 men with lower urinary tract symptoms doses of 50 mg selenium and 5 mg lycopene for 2 years, but no significant statistical differences were observed in terms of the risk of prostate cancer or the mean changes of PSA between the two groups. In a randomized controlled study by Gontero et al. [51], the outcomes of 60 patients with primary high-grade HGPIN or atypical small acinar proliferation (ASAP) were investigated. The patients received daily supplementation of lycopene (35 mg), selenium (55 µg), and GTCs (600 mg) for 6 months. After a follow-up period, 53 men underwent re-biopsy, and 13 of them (24.5%) were diagnosed with prostate cancer (PCa) (supplementation group: 10 cases, placebo group: 3 cases [*p* = 0.053]). This indicates that the administration of high doses of lycopene, GTCs, and selenium in men with HGPIN and/or ASAP was associated with a higher incidence of prostate cancer upon re-biopsy. These findings suggest the need for caution when considering the use of these co-complementary substances.

In this paper, we conducted a comprehensive analysis of selenium chemoprevention studies and further performed a subgroup analysis on different populations, as illustrated in Figure 2. The results indicate that selenium supplementation does not prove to be effective in preventing prostate cancer (HR: 0.95; 95% CI 0.80–1.13), neither for the general population (HR: 0.74; 95% CI 0.35–1.56) nor for those at high risk (HR: 0.95; 95% CI 0.71–1.26). This highlights that simply classifying the study population may not be enough, and it is crucial to consider other factors, such as the appropriate dosage of selenium supplementation and the influence of genetic background.

**Table 1:** Clinical trials using selenium as chemopreventive agents against prostate cancer.

Study	Type of Study	Initiation Year	Participants	Forms of Selenium Supplementation	Follow-Up	Preventive Effects for Prostate Cancer	HR/RR (95%CI)	References
Selenium supplementation in general population								
NPC	RCT	1983	927 men (457 selenium/470 placebo)	200 mg/day high selenium yeast	13 years	Positive	HR 0.48 (0.28, 0.80)	[43]
SELECT	RCT	2001	848 men (432 selenium/416 placebo)	200 µg/day selenized yeast/L-selenomethionine	Median follow-up 5.46 years	NS	HR 1.04 (0.83, 1.30)	[44]
Selenium supplementation in population with high risk of prostate cancer								
SWOG	RCT	2011	423 men with HGPIN (212 selenium and 211 placebo)	200 µg/d selenomethionine	3 years	NS	RR 0.97 (0.68–1.39)	[45]
NBT	RCT	2013	699 men with high risk of prostate cancer (467 selenium and 232 placebo)	200 µg/day selenium (N =234), or 400 µg/day selenium (N = 233)	5 years	NS	HR (selenium 200 µg/day) 0.94 (0.52, 1.7) HR (selenium 400 µg/day) 0.90 (0.48, 1.7)	[46]
ECOG 5597	RCT	2013	1561 men with a history of non–small-cell lung cancer (1040 selenium/521 placebo)	selenized yeast 200 µg/day	4 years	NS	RR 0.87 (0.39–1.95)	[47]
Selenium supplementation combined with vitamin E in general population								
SELECT	RCT	2001	853 men (437 selenium + vitamin E/ 416 placebo)	200 mg/d L-selenomethionine and 400 IU/d vitamin E	Median follow-up 5.46 years	NS	HR 1.05 (0.83, 1.31)	[44]
Procomb	RCT	2011	209 men with lower urinary tract symptoms (134 Lycopene + selenium and 75 placebo)	50 mg/d selenium and 5 mg/d lycopene	2 years	NS	HR 1.38 (0.32–5.90)	[52]

RCT = Randomized controlled study; HR = Hazard ratio; RR = Relative risk; NS = Non-significant.

SNPs in selenoproteins are believed to influence an individual’s response to selenium supplements. For instance, individuals with the AA and AG alleles in the TXNRD2 rs3804047 gene variant have shown an increased risk of prostate cancer with selenium supplementation. Conversely, individuals with the GG allele exhibited a preventive effect against high-grade prostate cancer risk with exogenous selenium supplementation. Similar trends were observed for the TXNRD2 rs8141691 variant, where individuals with the AA allele were less likely to develop high-grade prostate cancer risk with selenium supplementation, while those with the GG and AG alleles had an increased risk of high-grade prostate cancer [16]. These findings suggest that different genetic profiles respond differently to selenium supplementation in the general population, which may explain the varying effectiveness of selenium supplementation. Therefore, incorporating genetic factors into the determination of the appropriate population for prostate cancer chemoprevention can help maximize the potential benefits while minimizing the risks. Personalized approaches based on individual genetic profiles hold promise for optimizing the efficacy of selenium supplementation in reducing prostate cancer risk.

Several studies have explored the relationship between selenium concentration and prostate cancer risk. Waters et al. [53] outlined a U-shaped dose–response curve between toenail selenium concentration and prostatic DNA damage in dogs, suggesting that optimal selenium status may be more beneficial than excessively low or high levels. Gopalakrishna et al. [54] explained the U-shaped dose–response effects by elucidating how selenium binds to PKC at lower concentrations, inactivating the anti-apoptotic enzyme PKC and promoting the peroxyhydrogen reaction of tumor lipids. Conversely, high concentrations of selenium inactivate the pro-apoptotic enzyme, resulting in cell resistance to selenium-induced apoptosis. Additionally, Liu and colleagues [55] discovered that survivin can attenuate the inhibitory effects of selenium supplementation on cancer growth. Therefore, selecting patients with low survivin levels may enhance the benefits of selenium supplementation in prostate cancer.

In the field of research, the prevention of prostate cancer through chemoprevention has consistently been a crucial subject of discussion. Nevertheless, as per the latest studies, it has been established that selenium supplementation does not offer protection against prostate cancer in either healthy individuals or those at high risk. Screening the appropriate population for selenium supplementation in prostate cancer is a complex task that requires consideration of factors such as baseline selenium levels, age, overall health, genetic factors, and potential interactions with medications. Selenium intake should be carefully monitored to avoid excessive supplementation, as high doses can be toxic and may increase the risk of adverse effects.

## 4. Selenium and Prostate Cancer Therapy

Regarding the effects of selenium in cancer treatment, a study [56] sought to assess whether selenium supplementation could attenuate the progression of prostate cancer by administering daily doses of 0 µg, 200 µg, and 800 µg of selenium to prostate cancer patients, with PSA velocity used as a measurement parameter. The results revealed that selenium supplementation had no significant impact on PSA velocity in localized prostate cancer. Notably, high-dose selenium was observed to increase PSA velocity in men with elevated baseline plasma selenium concentrations, raising concerns about potential adverse effects from excessive supplementation in individuals with already elevated selenium levels. Additionally, another study by Stacey et al. [57] evaluated the mortality of 4459 patients who used selenium supplementation after diagnosis and found that supplementation of 140 μg/day or more following a nonmetastatic prostate cancer diagnosis increased prostate cancer mortality. Overall, based on the current studies, sole selenium supplementation does not demonstrate favorable outcomes in prostate cancer treatment. This could be attributed to the necessity of considering individual selenium levels, along with other contributing factors, and administering appropriate doses of selenium supplementation. It is worth noting that excessive supplementation may even have detrimental effects on the survival of prostate cancer patients.

Several studies have investigated the potential synergistic effects of selenium supplementation in combination with chemotherapy and radiotherapy for prostate cancer patients. Sodium selenite, for instance, has demonstrated a significant enhancement of the radiosensitizing effect in both HI–LAPC-4 and PC-3 xenograft tumors [58]. Additionally, another study [59] provided valuable evidence indicating that prostate cancer patients with low levels of selenium and lycopene are more susceptible to DNA damage induced by ionizing radiation. Husbeck et al. highlighted that selenite increases sensitivity to gamma radiation in prostate cancer by reducing the ratio of GSH:GSSG [60]. These findings suggest that selenium supplementation holds the potential to enhance the effects of radiotherapy in prostate cancer treatment. Moreover, pelvic radiotherapy has been linked to an increased incidence of radiotherapy-induced diarrhea in patients, and selenium supplementation has demonstrated the ability to decrease the risk of such diarrhea in selenium-deficient patients with uterine cancer [61].

In the context of chemotherapy, the combination therapy of docetaxel and sodium selenite has shown synergistic inhibitory effects on prostate cancer cell growth compared to using docetaxel alone [62]. Additionally, Tabassum et al. [63] suggested that antioxidant supplementation, which includes selenium, may have the potential to reduce the adverse effects of chemotherapy. Furthermore, selenium supplementation has been reported to decrease hormone therapy-related adverse effects [64].

In general, while selenium supplementation alone did not demonstrate a positive effect on prostate cancer progression, it shows promise in enhancing the efficacy of chemotherapy and radiotherapy while mitigating their associated side effects during cancer treatment. Consequently, conducting further studies and clinical trials is imperative to ascertain the optimal dosage, duration, and timing of selenium supplementation regimens.

## 5. Selenium-Related Protein in Prostate Cancer

### 5.1. Selenium-Containing Proteins

#### 5.1.1. Classification of Selenium-Containing Proteins

Selenium-containing proteins can be classified into two main types: selenoproteins, which incorporate selenium nonspecifically during translation, and selenium-binding proteins, which utilize selenium as a cofactor [65,66].

The first type comprises selenoproteins, where selenium exists as the amino acid selenocysteine and is integrated into the polypeptide chain through a distinct mechanism. These proteins play various essential roles in biological processes. Firstly, they act as antioxidant enzymes, such as GPX1, GPX2, GPX3, GPX4, GPX6, selenoprotein K (SELENOK), selenoprotein R (SELENOR), and selenoprotein W (SELENOW), contributing to the antioxidant system [67]. Secondly, there are proteins like TXRND1, TXRND2, and TXRND3 involved in redox signaling [68], as well as DIO1, DIO2, and DIO3, which play a role in thyroid hormone synthesis [69]. Additionally, there are other examples, such as SELENOP, responsible for selenium storage and transportation [70], and Selenoprotein F (SELENOF), Selenoprotein N (SELENON), and Selenoprotein M (SELENOM), which are involved in protein folding [71]. In general, these selenoproteins play critical roles in various cellular processes, which are presented in Table 2, including antioxidant defense, DNA repair, redox regulation, and modulation of cellular signaling pathways.

The second category of proteins includes those that do not incorporate selenocysteine (SeCys) encoded by UGA during their synthesis [72]. In mammals, only two proteins of this type have been identified: selenium-binding protein 1 (SBP1) and selenium-binding protein 2. Of particular interest is SBP1, although its precise biological function remains uncertain. In an attempt to elucidate its role, a clinical study investigated the nucleoplasm-to-plasma ratio of SBP1 in tissue arrays obtained from 202 patients with recurrent prostate cancer and 202 matched non-recurrent prostate cancer cases [73]. The study revealed that higher nuclear SBP1 levels and a greater nuclear-to-cytoplasm ratio were associated with lower tumor grade. Interestingly, tumors in the lowest quartile of SBP1 levels were found to have over twice the likelihood of relapse compared to tumors in any other quartile. This suggests a potential involvement of SBP1 in the development of prostate disease.

**Table 2:** Summary of Selenoprotein Functions.

Selenoproteins	Functions	References
GPX1	Metabolize hydrogen peroxide and some organic hydroperoxides	[74]
GPX2	Antioxidant activity in gastroin testinal tissues	[74]
GPX3	Reduce H_2_O_2_, fatty acid hydroperoxides, and phospholipid hydroperoxides in the plasma and thyrocytes	[74]
GPX4	Reduce phospholipid- and cholesterol-hydroperoxides by using GSH	[74]
GPX6	Reduce olfactory organs H_2_O_2_	[74]
TXNRD1	Antioxidant activity and regenerate reduction of thioredoxin	[68]
TXNRD2	Regenerates reduced thioredoxin in mitochondria	[68]
TXNRD3	Redox regulation	[68]
DIO1	Production of T3 in thyroid and peripheral tissues	[69]
DIO2	Production of T3 in peripheral tissues	[69]
DIO3	Inactivates thyroid hormone	[69]
SELENOK	Antioxidant activity	[75]
SELENOR	Antioxidant activity and protein repair	[76]
SELENOW	Antioxidant role	[77]
SELENOP	Secreted into plasma for selenium transport to tissues	[70]
SELENOF	Correcting misglycosylated/misfolded glycoproteins	[71]
SELENON	Its mutation leads to early-onset myopathies	[78]
SELENOM	Antioxidant activity	[71]
SELENOS	Deletes the misfolded proteins in endoplasmic reticulum and responds to endoplasmic reticulum stress	[79]
SELENOI	Involved in phospholipid biosynthesis	[80]
SELENOT	Oxidoreductase involved in redox regulation and cell anchorage	[81]
SELENOO	Unknown	[65]
SELENOV	Unknown	[65]
SELENOH	Redox sensing and transcriptional regulation of glutathione	[82]

#### 5.1.2. Anti-Tumor Role of Selenium-Containing Proteins

An essential mechanism by which selenium-containing proteins exert their anti-prostate cancer effects is through the regulation of redox homeostasis.

Selenoproteins like GPXs and TXNRDs are powerful antioxidants that play a crucial role in cellular protection. These antioxidant enzymes catalyze the reduction of hydrogen peroxide, lipid hydroperoxides, and other organic hydroperoxides by utilizing GSH, effectively shielding cells from oxidative damage [83]. By maintaining redox balance, selenoproteins help prevent DNA mutations and genomic instability, both of which are associated with the development and progression of prostate cancer [84]. Numerous studies have demonstrated an imbalance in oxidative stress/redox status and a compromised GPX/Thiol antioxidant system in prostate cancer patients, accompanied by reduced activities of superoxide dismutase (SOD), GPX, GSH, and catalase (CAT) compared to controls. Specifically, a study including 222 prostate cancer patients, 67 BPH patients, and 23 control subjects showed that prostate cancer patients had significantly lower levels of total -SH, GPX, and GSH, and the combination of -SH, GPX, and GSH when compared to healthy controls [84,85]. Additionally, research investigating the mRNA profile of DU145 cells treated with sodium selenite observed an increased expression of GPX1, GPX2, and GPX4 and a decreased expression of TXNRDs in prostate cancer [86]. This means that not only do oxidoreductase abnormalities exist in prostate cancer, but selenium supplementation can also alter the oxidoreductase-related enzymes to change this imbalance.

Elhodaky and colleagues discovered that SBP1 plays a significant role in regulating the energy metabolic balance through oxidative phosphorylation in prostate cancer cells. This is achieved by producing H_2_O_2_, which helps maintain the cells’ ability to metastasize [87]. Moreover, it has been established that SBP1 and GPX-1 interact with each other, and their protein levels have an inverse relationship. When GPX-1 enzyme activity increases, SBP1 levels decrease. Conversely, elevated SBP1 weakens GPX-1 activity, but it does not affect mRNA levels [88,89]. These findings suggest that the interaction between selenoproteins influences enzyme activity, raising the possibility that GPX1 could hinder prostate cancer cells’ metastatic potential by regulating SBP1.

Furthermore, SELENOP is reported to be downregulated in 68% of prostate cancer tissues. Knockdown of SELENOP expression in prostate cancer cells resulted in increased production of ROS and attenuated cell viability in vitro [90]. These findings suggest that a deficiency or downregulation of selenium proteins can result in an imbalance in oxidative stress and antioxidant levels, leading to the accumulation of oxidative stress products in prostate tumor tissues. Intriguingly, Luchman and colleagues [91] observed that mice deficient in the SeCys tRNA gene Trsp exhibited increased lipid peroxidation and developed extensive HGPIN at 6 weeks, followed by high-grade dysplasia and microinvasive carcinoma at 24 weeks, even without knocking down other genes. Similarly, the loss of GPX3 in Nk3.1 knock-out mice induces oxidative stress, thereby enhancing hyperplasia of intraepithelial neoplasia in the pre-cancerous stage of the prostate, independent of the Wnt/β-catenin signaling pathway [92]. These observations highlight the importance of maintaining adequate levels of selenium and functional selenoproteins to mitigate oxidative damage and prevent the early stages of prostate cancer, supporting the inhibitory role of selenoproteins in the development and progression of prostate cancer.

Abnormal lipid metabolism is a key characteristic of prostate cancer, and a study by Chang et al. [93] found a high intake of animal fat enhances prostate cancer progression in a murine model by suppressing GPX3 expression and increasing the proliferation of prostate intraepithelial neoplasia epithelial cells. This means incorporating a balanced diet with a focus on healthier fats and increased intake of antioxidant-rich foods may help mitigate the risk of prostate cancer and its progression. Exploiting microRNA molecules, such as miR-17, to target antioxidant enzymes is involved in prostate cancer cell proliferation, and it has been reported that increasing miR-17 inhibits the growth of PC-3 cells by eliminating critical primary mitochondrial antioxidant enzymes, such as manganese superoxide dismutase (MnSOD), GPX2, and TXNRD2 [94]. This knowledge can guide the development of targeted interventions that specifically affect the relevant cellular compartments, optimizing the efficacy of antioxidant therapies in preventing or treating prostate cancer.

Endoplasmic reticulum (ER) stress, also denoted as the “unfolded protein response (UPR)”, is a cellular mechanism characterized by the accumulation of misfolded proteins within the endoplasmic reticulum. The prevailing consensus posits that adaptive ER stress is generally heightened in tumors [95,96]. Knocking down the UPR response factor XBP1 has been found to impede tumor cell growth [97], signifying the indispensability of UPR in maintaining ER homeostasis and the survival of cancer cells under specific conditions. However, excessive oxidative stress that disrupts the balance can induce ER stress and trigger tumor cell apoptosis. The application of methylseleninic acid (MSeA) has been documented to instigate ER stress through the activation of the PERK signaling pathway in DU145 cells, leading to the upregulation of apoptosis-related genes, including caspase-3 and caspase-4 [98]. These findings collectively suggest that UPR is critical for tumor growth. Conversely, an excessive external stimulus can overwhelm UPR recovery, activating apoptotic signals and potentially exerting anti-tumor effects.

Numerous selenoproteins localized within the ER, such as SELENOT, SELENOM, SELENOK, SELENOS, SELENON, and DIO2, have been identified to participate in the biological processes associated with ER stress across breast cancer, prostate cancer, and fibrosarcoma [96]. Previous studies have also shown that the treatment of DU145 cells with 1 µM MSeA for 24 h led to a significant upregulation in the gene expression of SELENOF and SELENOM [97], suggesting a potential role in regulating exogenously induced ER stress and exerting anti-tumor effects. The augmentation of selenium protein expression was also observed, with the induction of ER stress subsequent to the treatment of prostate cancer cells with selenium nanoparticles [99]. Furthermore, selenoprotein T has been highlighted as a crucial adaptive molecule facilitating the accumulation of reactive oxygen species during stress induced by high endocrine cell hormone levels [100]. Additionally, cells overexpressing SELENOV exhibited enhanced redox enzyme activities and reduced expressions of ER stress-associated markers [101], ultimately affording selenoprotein V a protective role against reactive oxygen-mediated ER stress-related signaling. Collectively, these findings underscore the potential anti-tumor capacity of selenoproteins in regulating endoplasmic reticulum stress, but the optimal concentration that balances adaptation and excess ER stress remains ambiguous. Furthermore, the engagement of distinct selenium proteins in ER stress regulation might exhibit tissue-specific characteristics.

Several studies have investigated the association between SNPs of selenoproteins and the risk of overall and advanced prostate cancer. In our review, we identified several SNPs of SEPP1, GPX1, GPX2, GPX4, and TXNRD2 that are related to the risk of prostate cancer in Table 3. Chan et al. [16] identified 130 SNPs in 21 genes from the SELECT cohort study and revealed an association between SNPs of CAT, GPX1, SOD1, SOD2, SOD3, TXNRD2, SEC14L2, and TTPA, and the risk of high-grade prostate cancer. Notably, for TXNRD2, individuals carrying different alleles responded differently to selenium supplementation. Méplan reviewed several SNP studies of selenoproteins and their relationship with prostate cancer and found that serum selenium and selenoprotein concentrations were related to prostate cancer only when the allele of TXNRD2 rs9605031 was CT/TT [102]. This is consistent with the results of a prospective study in European men, where individuals with the CT/TT allele of GPX1 rs1050450 showed a protective effect of serum selenium on prostate cancer [15]. Assessing selenoprotein SNPs in individuals may help identify those at higher risk of prostate cancer. Genetic screening could contribute to personalized risk assessment and enable targeted interventions or monitoring for individuals with specific genetic variations. Moreover, selenoprotein SNPs can modify the risk of prostate cancer and are associated with risk factors such as Gleason score, stage, grade, etc. Cooper et al. [103] revealed that men homozygous for selenoprotein P-Ala234 who were also SOD2-Ala16+ had a higher risk of prostate cancer, and this interaction was stronger in ever-smokers, where the ability to remove increased mitochondrial H_2_O_2_ is compromised, thereby promoting proliferation and migration of prostate tumor cells. This suggests that recognizing the interactions between selenoprotein SNPs and environmental factors, such as smoking, highlights the importance of considering gene–environment interactions in prostate cancer risk assessment.

Overall, selenoproteins have been implicated in the modulation of antioxidant properties and endoplasmic reticulum stress involved in prostate cancer, and selenoprotein SNPs are reported to have a correlation with the risk of prostate cancer. Tailoring interventions based on individual genetic profiles may improve efficacy and reduce adverse effects. Exploiting the therapeutic potential of selenoproteins could lead to the development of novel strategies for prostate cancer therapy.

### 5.2. Selenium in the Development and Progression of Prostate Cancer

**Figure 3:**
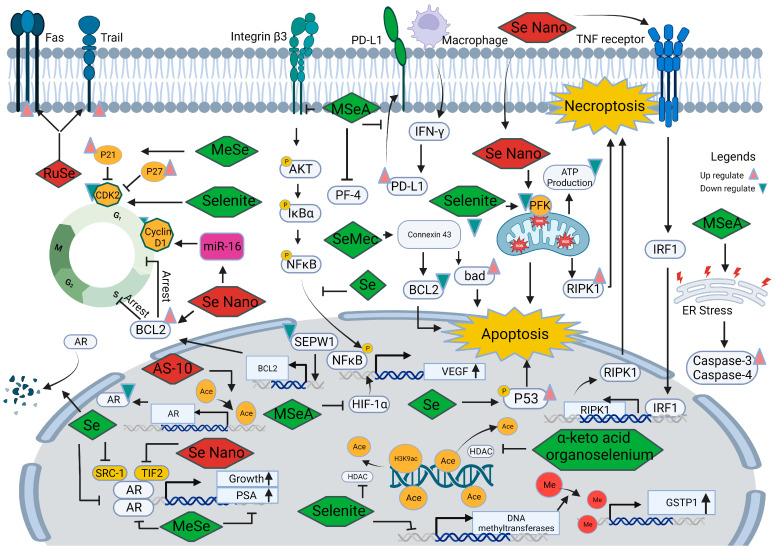
Schematic representation of selenium’s roles in the development and progression of prostate cancer.

Further research is essential to better understand the mechanisms underlying the contradictory findings on relationships associated with selenium and prostate cancer. Figure 3 illustrates multiple interconnected pathways through which selenium is believed to exert its influence in the development and progression of prostate cancer. These pathways include the modulation of oxidative stress, inflammation, angiogenesis, androgen signaling, ferroptosis, epigenetic modulation, immune responses, and interactions with other trace elements and minerals. Comprehensive knowledge of these intricate interactions has the potential to unlock new possibilities for targeted interventions and the development of personalized approaches in the prevention and treatment of prostate cancer.

#### 5.2.1. Selenium and Androgen Receptor

The androgen receptor (AR) signaling pathway plays a crucial role in the development of prostate cancer. Agents that block this pathway, such as abiraterone and enzalutamide, have proven to be effective treatments, significantly extending the survival of prostate cancer patients [107]. A proteomic profile study conducted on a transgenic prostate cancer mouse model exposed to methyl-selenium compounds revealed changes in proteins associated with prostate functional differentiation, androgen receptor signaling, protein folding, and endoplasmic reticulum stress responses [108]. Consistent with these findings, monomethylated selenium (MeSe) has been shown to reduce the growth of LNCaP human prostate cancer xenografts, accompanied by a decrease in androgen receptor and prostate-specific antigen expression [109]. Furthermore, Chun et al. [110] observed that selenium increased the degradation of AR protein and reduced its nuclear localization in LNCaP cells while not affecting ligand binding to AR. Selenium also influences the recruitment of co-activated molecules, such as SRC-1 and TIF-2, in the promoter region of AR binding genes, disrupting the normal signaling pathway. These collective results indicate that selenium can disturb AR signaling by decreasing its transcriptional and translational levels and reducing the ability of target gene binding.

Recent advancements have explored novel drugs that target the androgen receptor signaling pathway to inhibit the growth of prostate cancer. Kong et al. [111] demonstrated that selenium nanoparticles can suppress the growth of prostate cancer cells by disrupting the androgen receptor. Another promising compound, AS-10, a seleno-aspirin derivative, was found to suppress androgen receptor expression by promoting histone acetylation [112]. When combined with conventional androgen receptor inhibitors, AS-10 showed synergistic effects in prostate cancer treatment.

In the context of supplementation with multiple microminerals, interactions can occur at various levels, including absorption, competition for binding sites, and metabolic pathways. An in vivo study on Wistar rats investigated the expression of AR following the administration of selenium and zinc, where additional selenium supplementation weakened the effect of zinc on the increase in AR expression in the prostate [11]. Therefore, when designing supplementation regimens, it is crucial to consider interactions between microminerals and the impact of selenium supplementation on the effects of other micronutrients, such as zinc, and should be carefully evaluated to avoid potential interference with desired outcomes.

#### 5.2.2. Selenium and Cell Cycle

Dysregulation of cyclin-dependent kinases (CDKs), cyclins, and CDK inhibitors plays a significant role in the process of cancer development. In prostate cancer, abnormal cell cycle control is often observed, resulting in uncontrolled cell growth and division [113]. Analysis of DNA microarrays revealed that among the selenoproteins, SEPW1 was the only one observed to increase in prostate epithelial cells treated with 100 nM sodium selenite [114]. Knocking down SEPW1 in prostate epithelial cells induced G1 arrest by increasing BCL2 mRNA expression and reducing transcripts related to S-phase and G2/M-phase genes, leading to blockage of G1/S transition. These findings suggest that selenoprotein may prevent cell cycle arrest and help maintain normal prostatic function.

Furthermore, treatment with methyl selenium or selenite can induce cell cycle arrest in DU145 cells by upregulating CDK inhibitors, such as p27kip1 and p21cip1, and downregulating CDK2 [12]. Wang et al. [115] pointed out that G1 arrest induced by methylseleninic acid (MSeA) in DU145 cells can be reversed by knocking down p21. Similarly, persistent exposure to a seleno-aspirin compound predominantly arrested prostate cancer cells in the G1 phase. These results collectively indicate that both organic and inorganic forms of selenium primarily regulate G1 arrest in prostate cancer cells by regulating CDK and CDK inhibitors.

New selenium drugs are being used to enhance the anti-tumor effects of conventional drugs by cell cycle arrest. The combined treatment of zoledronic acid and lentinan-functionalized selenium nanoparticles has been shown to reduce prostate cancer cell viability. This effect may be attributed to the regulation of BCL2 expression, resulting in irreversible DNA damage and induction of S-phase arrest [13]. Selenium nanoparticles have also been found to upregulate miR-16 [116], which reduces the expression of cyclin D1 and BCL-2, leading to enhanced apoptosis induction in prostate cancer cells.

In summary, the involvement of cell cycle regulators and the BCL2 protein family in cell cycle arrest and apoptosis induced by selenium supplementation in prostate cancer is evident. A range of forms of selenium can target cell cycle regulatory proteins to arrest tumor cells in the G1/S phases but not G2/M phase; it has been demonstrated by Zhao et al. that selenite supplementation alone can’t induce G2/M phase blockage [117]. Understanding the molecular mechanisms underlying cell cycle dysregulation provides valuable insights into the pathogenesis of prostate cancer and identifies potential therapeutic targets for the development of novel treatment strategies.

#### 5.2.3. Selenium and Angiogenesis

Aberrant blood vessel formation is a common characteristic of cancer [118]. Tumor cells have the ability to secrete high levels of pro-angiogenic factors, including vascular endothelial growth factor (VEGF), platelet-derived growth factor (PDGF), and transforming growth factor (TGF), which contribute to the development of an abnormal vascular network. VEGF plays a crucial role in prostate cancer angiogenesis, and excessive VEGF promotes the continuous growth of prostate cancer. Preliminary data [119] suggest that microvessel density increases in higher-grade prostate cancer and can serve as a predictor of worse outcomes.

Feeding mice harboring orthotopic PC-3 tumors with drinking water containing different forms of selenium (sodium selenate, SeMet, methylselenocysteine, and selenized yeast), inorganic selenium can suppress the growth of prostate cancer partially due to angiogenesis inhibition [120]. MSeA has been observed to delay the mitogen-stimulated progression of telomerase-immortalized microvascular endothelial cells from G1 to S phase, and daily oral MSeA treatment of nude mice bearing DU145 xenografts inhibited tumor growth in a dose-dependent manner, decreasing tumor microvessel density in cancer tissue [121]. It has also been reported [122] that MSeA downregulates integrin β3 and disrupts its clustering, thereby delaying the angiogenesis process through reduced phosphorylation of AKT, IκBα, and NFκB. Similarly, in a prostatitis-induced cancer model, low doses of selenium can inhibit the nuclear translocation of NFκB and the subsequent production of the pro-angiogenic factor VEGF in PC-3 cells. Overall, selenium and MSeA can disrupt vascular formation in cancer tissues, where VEGF and NF-(K)B are responsive factors to selenium supplementation.

Hypoxia-inducible factor-1α(HIF-1α), a transcription factor increased under hypoxia conditions, can induce a variety of downstream pro-proliferative cytokines expression, such as VEGF, thereby promoting tumor growth. Additionally, studies have shown that MSeA treatment reduces hypoxia-inducible factor-1α in invasive prostate cancer, thereby suppressing the growth of hormone-refractory prostate cancer [123]. Micronutrient supplementation has also been found to attenuate prostate cancer progression by inducing the expression of platelet factor-4, a megakaryocyte-specific inhibitor of angiogenesis [124]. These findings indicate that selenium likely modulates angiogenesis in prostate cancer through VEGF, hypoxia-inducible factor (HIF), and platelet factor-4 (PF4).

Few clinical trials [124] targeting the VEGF pathway among prostate cancer patients showed favorable survival outcomes with acceptable toxicity. Selenium and its compounds have been shown to interact with angiogenesis modulation factors. Therefore, combining selenium supplementation with VEGF-targeted drugs or other anti-angiogenic therapies could potentially enhance their efficacy. Selenium’s ability to modulate angiogenesis-related factors suggests that it may complement existing treatment strategies by strengthening the anti-angiogenic effects and inhibiting prostate cancer growth.

#### 5.2.4. Selenium and Cell Death

Cell death is a crucial process in various biological functions, including development, tissue homeostasis, and the elimination of damaged cells. Selenium and selenoproteins have been shown to play a role in different types of cell death, such as apoptosis, ferroptosis, and necroptosis, with potential implications for prostate cancer development, progression, and treatment response.

Apoptosis, a highly regulated process known as programmed cell death, is influenced by selenium in prostate cancer. Selenium and its compounds can exert anti-tumor effects by modulating apoptosis through various mechanisms. Selenium’s involvement in apoptosis is associated with G1 and S phase arrest as well as the AR signaling pathway [125]. The p53, a tumor suppressor gene, has been studied, and it plays a critical role in preventing the development and progression of cancer. Further, selenium can trigger rapid transcriptional activation of phosphorylation of p53 in LNCaP cells accompanied by increased protein levels of several p53 target genes, such as p21, Bax, DR5, and PIG-3, to induce apoptosis. Blocking p53 function significantly reduces selenium-induced apoptosis, highlighting the critical role of p53 in tumor cell apoptosis induced by selenium [126]. Additionally, selenium compounds like se-methylselenocysteine (SeMec) can activate connexin 43 at the transcriptional and protein level, thereby downregulating BCL-2 and upregulating bad expression, contributing to cell apoptosis [127]. Proteomics analysis of PC-3 cells exposed to selenite revealed significant changes in proteins related to mitochondrial pathways, endoplasmic reticulum stress, and HIF-1α mediated pathways, suggesting the importance of mitochondrial and endoplasmic reticulum functions in H2Se-induced apoptosis [98]. Similarly, selenite treatment has been found to induce cell apoptosis through the mitochondrial-dependent pathway and improve superoxide production, which can be suppressed by overexpression of MnSOD [128]. Based on the findings from Varlamova et al. [99], it was observed in DU145 cells that NanoSe exhibited a dose-dependent inhibition of cellular activity, selectively impacting cancer cells while not influencing the activity of normal connective tissue cells. This phenomenon could be attributed to the upregulation of pro-apoptosis genes, including BIM, PUMA, BAK, and BAX. Taken together, selenium can induce apoptosis by various mechanisms, including G1 and S phase arrest, activation of p53 and its target genes, downregulation of BCL-2, upregulation of pro-apoptotic proteins, mitochondrial pathway modulation, and endoplasmic reticulum stress.

Necroptosis is a programmed form of necrosis characterized by mitochondrial alterations and plasma membrane permeabilization. It is regulated by kinase enzymes, such as inorganic selenium. According to Cui et al. [129], selenite trigger a decrease in phosphofructokinase activity and ATP depletion induce necroptosis in PC-3 cells, can be counteracted by serine/threonine protein kinase (RIPK) and mixed lineage kinase domain like pseudokinase (MLKL) inhibitor. Biogenic selenium nanoparticles have demonstrated higher anti-cancer activity and lower toxicity in murine models of prostate cancer [130]. These nanoparticles have been found to increase ROS levels in mitochondria or bind to the TNF receptor, resulting in elevated expression of RIPK1 and the formation of necrosomes in PC-3 cells. The effects induced by the nanoparticles can be reversed by the use of necroptosis inhibitors [131]. Together, mitochondrial dysfunction and necroptosis factor RIPK1 are necessary for selenium-induced necroptosis, and this holds potential as a therapeutic approach in prostate cancer treatment.

Ferroptosis is a recently identified form of regulated cell death characterized by the iron-dependent accumulation of lipid peroxides and oxidative stress. GPX4, a selenoprotein, plays a role in preventing lipid peroxide toxicity and maintaining the homeostasis of the membrane lipid bilayer, thus inhibiting ferroptosis through its enzymatic activity. Inhibitors of GPX4, such as RSL3, have exhibited inhibitory effects on treatment-resistant prostate cancer in vivo without measurable side effects [132]. Combination therapies involving RSL3 and second-generation antiandrogens have also shown promising results in halting prostate cancer cell growth in vivo [133]. Selenium supplementation increases GPX4 expression and confers resistance to ferroptosis induction in cancer cells [134]. Additionally, normal prostate epithelium has been found to have higher selenium bioavailability compared to prostate cancer, suggesting that prostate cancer cells have poor antioxidant capacity, leading to the accumulation of oxidative stress products and impaired function [135]. Overall, the association between ferroptosis and selenium in prostate cancer highlights the potential importance of selenium in regulating cell death pathways and maintaining cellular homeostasis.

In summary, selenium’s involvement in different types of cell death pathways presents intriguing possibilities for understanding and treating prostate cancer. Further investigation is required to elucidate the underlying mechanisms.

#### 5.2.5. Selenium and Epigenetic Modifications

Epigenetics is a mechanism that can modulate gene expression without altering the DNA sequence of the genome. It encompasses various genetic modifications, including DNA methylation, histone modifications, chromatin remodeling, and non-coding RNA molecules, all of which can profoundly influence gene expression and cellular behavior [136]. Previous studies have explored the epigenetic regulation of selenium proteins and selenium compounds in cancer [137].

In LNCap cells, the treatment with selenite induces DNA demethylation of the glutathione-S-transferase (GSTP1) promoter by reducing mRNA levels of DNA methyltransferases, ultimately leading to the upregulation of GSTP1 protein expression. Additionally, selenite treatment decreases histone deacetylase activity and increases the levels of acetylated lysine 9 on histone H3 [138]. On the other hand, the methylation status of GSTP1 and Ras-associated family 1A genes remains unaltered following seleno-DL-methionine and selenium treatment in prostate cancer cells [139]. These findings suggest that different forms of selenium may have varying effects on epigenetic modifications in prostate cancer. Furthermore, the alpha-keto acid metabolites of organoselenium compounds share structural similarities with HDAC inhibitors like butyrate, enabling them to modulate H2DAC activity and histone acetylation status [140].

As mentioned earlier, the involvement of miR-16 and miR-17 was observed in the regulation of cyclin D1 expression and selenoprotein in the antioxidative system. These regulatory actions had inhibitory effects on tumor proliferation, promoted apoptosis induction, and maintained the balance of redox status in prostate cancer cells.

These findings provide compelling evidence that selenium can influence DNA methylation, histone modifications, and HDAC activity, highlighting its potential as an epigenetic regulator in the development of prostate cancer.

#### 5.2.6. Other Mechanisms

In addition to the mechanisms discussed above, there are additional factors that contribute to our understanding of the relationship between selenium and prostate cancer, namely epithelial–mesenchymal transformation (EMT) and immunomodulation.

Epithelial–mesenchymal transformation (EMT) is a biological process involved in embryogenesis and tissue repair. However, abnormal activation of EMT has been implicated in cancer metastasis and invasion. A recent study [141] compared the transition zone in prostate biopsies from men who received 300 µg of selenium per day in the form of selenized yeast (*n* = 12) to those who received a placebo (*n* = 11) over a 5-week period. The study found that selenium intervention upregulated E-cadherin and epithelial cell adhesion molecule EPCAM, which are epithelial markers while downregulating the mesenchymal markers vimentin and fibronectin. This suggests that selenium compounds may possess significant anti-EMT properties in prostate cancer cells.

The abnormal immune state of the tumor microenvironment has gained considerable attention in recent research, and immunotherapy has emerged as a promising treatment approach for cancer. Selenium supplementation has been reported to enhance immune cell activity, improve lymphocyte function, and increase cytokine production, thereby influencing the immune system’s ability to recognize and eliminate cancer cells [142]. Pretreating PC-3 cells with a selenium-bearing ruthenium complex (RuSe) has been shown to enhance their sensitivity to natural killer (NK) cell killing by increasing the expression of Trail and Fas in prostate cancer cells. In vivo studies have demonstrated that RuSe inhibits prostate tumor growth, indicating its potential as a highly effective immune sensitizer in immunotherapy [143]. Additionally, MSeA has been found to suppress IFN-γ-induced programmed death-ligand 1 (PD-L1) expression in prostate cancer, which can reverse PD-1/PD-L1 inhibitor resistance induced by cisplatin [144]. By enhancing immune cell activity, improving lymphocyte function, and influencing the expression of immune checkpoints, selenium can contribute to a more favorable immune response against prostate cancer cells.

Overall, leveraging the effects of selenium on the immune system and EMT holds great promise for the development of novel anti-cancer therapies. Further research and clinical trials are necessary to fully understand the optimal utilization of selenium in these therapeutic strategies.

## 6. Perspectives and Future Directions

The relationship between selenium and prostate cancer has garnered significant attention in research and clinical practice. While numerous studies have provided valuable insights, there are still important perspectives and future directions that warrant exploration to advance our understanding and optimize the potential of selenium in the prevention and treatment of prostate cancer.

Contrary to expectations, most clinical trials did not show any beneficial effect of selenium on prostate cancer chemoprevention or even a harmful effect. This alerts us that selenium exhibits its effect on prostate cancer based on very complex mechanisms. Determining the optimal dosage and duration of selenium supplementation is essential for achieving desired therapeutic effects. Integrating genomic, epigenomic, and proteomic profiling may enable the development of precision medicine approaches, allowing for tailored selenium interventions based on an individual’s genetic and molecular characteristics. Future studies should investigate dose–response relationships, considering variations in individual selenium status, genetic factors, and disease stage.

Combining selenium supplementation with other therapeutic modalities holds promise for enhanced treatment outcomes in prostate cancer. Exploring synergistic effects with conventional treatments, such as chemotherapy, radiotherapy, and hormone therapy, may lead to improved therapeutic responses and reduced side effects. More preclinical and clinical trials are warranted to determine the most effective combination regimens and the optimal sequencing of treatments.

Considering the interplay between selenium and other dietary factors, such as vitamins, minerals, and phytochemicals, could provide stronger preventive effects than selenium alone in prostate cancer, and as reviewed earlier, additional selenium supplementation weakened the stimulating effect of zinc on AR expression in the prostate. Therefore, investigating the synergistic effects of selenium with other bioactive compounds may uncover novel nutritional strategies for prostate cancer prevention and treatment.

Several mechanisms have been proposed to explain the association between selenium and prostate cancer, including cell death, cell cycle, angiogenesis, oxidative stress, AR signaling and epigenetic modifications, immunoregulation, and epithelial–mesenchymal transformation. However, there is still much to uncover in these fields. Further investigation is required to elucidate the intricate molecular pathways through which selenium exerts its anti-cancer effects. This understanding will not only deepen our knowledge of prostate cancer biology but also pave the way for the development of novel therapeutic targets and interventions.

Numerous agents, including selenium-containing small molecules such as selenium-bearing ruthenium complex, se-nanoparticles, and seleno-aspirin derivative, have been widely investigated in prostate cancer therapy, where they exhibit anti-tumour development and immune system-related effects with higher efficacy and lower toxicity. Therefore, exploiting novel therapeutic agents based on their unique properties and mechanisms of action to improve treatment outcomes and potentially overcome treatment difficulties, such as castration resistance in prostate cancer, is promising.

## 7. Conclusions

In summary, selenium exhibits diverse properties that make it a promising trace mineral in the realm of prostate cancer prevention, development, treatment, and the exploration of novel therapeutic agents. While the level of selenium in the body is associated with prostate cancer incidence and progression, conventional selenium supplementation does not seem to yield the expected impact on prostate cancer in the general population (HR: 0.95; 95% CI 0.80–1.13). This discrepancy may be attributed to various factors, including genetic variations, selenium usage, and individual selenium levels. In the context of combinatorial therapy, selenium has demonstrated promising synergistic potential in the treatment of prostate cancer. Previous research has indicated that selenium influences prostate cancer through various pathways, encompassing AR signaling, antioxidative properties, cell death, cell cycle regulation, angiogenesis, epigenetic regulation, immunoregulation, epithelial–mesenchymal transformation, and redox signaling. Collaborative efforts among researchers, clinicians, and patients are essential for maximizing the potential benefits of selenium in prostate cancer and translating research findings into clinical practice.

## Figures and Tables

**Table 3:** The risk of prostate cancer and response to selenium supplementation is modified by selenium protein single nucleotide polymorphism.

Gene	SNP ID	Risk of Prostate Cancer	Response to Selenium Supplementation	References
SELENOP	rs13168440	NS	NA	[104]
		TT decreases the risk of prostate cancer, compared to allele CC	NA	[105]
	rs230813	NS	NA	[104]
	rs230819	NS	NA	[104]
	rs3877899	NS	NA	[104]
		AA decreases the risk of distant prostate cancer, compared to allele GG	NA	[106]
	rs7579	AG and AA increase the risk of advanced (Stage III, IV) prostate cancer, compared to allele GG	NA	[104]
		NS	NA	[15]
	rs3797310	TT increases the risk of distant prostate cancer, compared to allele CC	NA	[106]
	rs3877899	NS	NA	[15]
	rs11959466	NS	NA	[105]
	rs12517112	NS	NA	[105]
	rs230820	NS	NA	[105]
SELENO15	rs561104	NS	NA	[106]
	rs540049	NS	NA	[15]
	rs5859	NS	NA	[15]
SELENOK	rs9880056	NS	NA	[102]
GPX1	rs17650792	AG and GG decrease the risk of high-grade prostate cancer, compared to allele AA	NA	[16]
		GG and AG increase the risk of high-grade (Stage III, IV) prostate cancer, compared to allele AA	NA	[104]
	rs1800668	TT and CT decrease the risk of high-grade (Stage III, IV) prostate cancer, compared to allele CC	NA	[104]
	rs3448	NS	NA	[104]
		TT decreases the risk of overall (local and distant) prostate cancer, compared to allele CC	NA	[106]
	rs1050450	NS	NA	[15]
GPX2	rs4902346	GG increases the risk of Gleason 7–10 prostate cancer, compared to allele AA	NA	[106]
GPX3	rs8177447	NS	NA	[106]
GPX4	rs2075710	TT increases the risk of local prostate cancer, compared to allele CC	NA	[106]
	rs713041	NS	NA	[15]
TXNRD1	rs7310505	NS	NA	[102]
TXNRD2	rs3804047	NA	Selenium supplementation increases the risk of high-grade prostate cancer in candidates with allele AA, AG	[16]
			Selenium supplementation decreases the risk of high-grade prostate cancer in candidates with allele GG	[16]
	rs8141691	AG and AA increase the risk of high-grade prostate cancer, compared to allele GG.	Selenium supplementation increases the risk of high-grade prostate cancer in candidates with allele AA, GG	[16]
			Selenium supplementation decreases the risk of high-grade prostate cancer in candidates with allele AA	[16]
	rs9605030	NS	NA	[102]
	rs9605031	NS	NA	[102]

Abbreviation: NS = Non-Significant; NA = Not Mentioned; GPX = Glutathione Peroxidase; TXNRD = Recombinant Thioredoxin Reductase; SELENOP = Selenoprotein P; SELENOK = Selenoprotein K.

## Data Availability

Data and materials can be provided upon request from the corresponding author.
